# Curcumin and Quercetin-Loaded Nanoemulsions: Physicochemical Compatibility Study and Validation of a Simultaneous Quantification Method

**DOI:** 10.3390/nano10091650

**Published:** 2020-08-22

**Authors:** Gustavo Richter Vaz, Adryana Clementino, Juliana Bidone, Marcos Antonio Villetti, Mariana Falkembach, Matheus Batista, Paula Barros, Fabio Sonvico, Cristiana Dora

**Affiliations:** 1 Laboratório de Nanotecnologia Aplicada à Saúde, Programa de Pós-Graduação em Ciências da Saúde, Universidade Federal do Rio Grande, Rio Grande 96210-900 (RS), Brazil; richtervaz@gmail.com (G.R.V.); mari_falkembach@hotmail.com (M.F.); mbmatheus54@gmail.com (M.B.); alicebarros.pb@gmail.com (P.B.); 2 Coordenação de Aperfeiçoamento de Pessoal de Nível Superior (CAPES), Brasilia 70040-020 (DF), Brazil; 3 Food and Drug Department, University of Parma, 43124 (PR) Parma, Italy; adryanarc@gmail.com; 4 Centro de Ciências Químicas, Farmacêuticas e de Alimentos, Universidade Federal de Pelotas, Pelotas 96010-900 (RS), Brazil; julianabidone@gmail.com; 5 Laboratório de Espectroscopia e Polímeros, Departamento de Física, Universidade Federal de Santa Maria, Santa Maria 97105-900 (RS), Brazil; mvilletti@hotmail.com

**Keywords:** HPLC method, curcumin, quercetin, thermal analysis, nanoemulsion

## Abstract

Biphasic oil/water nanoemulsions have been proposed as delivery systems for the intranasal administration of curcumin (CUR) and quercetin (QU), due to their high drug entrapment efficiency, the possibility of simultaneous drug administration and protection of the encapsulated compounds from degradation. To better understand the physicochemical and biological performance of the selected formulation simultaneously co-encapsulating CUR and QU, a stability test of the compound mixture was firstly carried out using X-ray powder diffraction and thermal analyses, such as differential scanning calorimetry (DSC) and thermogravimetric analyses (TGA). The determination and quantification of the encapsulated active compounds were then carried out being an essential parameter for the development of innovative nanomedicines. Thus, a new HPLC–UV/Vis method for the simultaneous determination of CUR and QU in the nanoemulsions was developed and validated. The X-ray diffraction analyses demonstrated that no interaction between the mixture of active ingredients, if any, is strong enough to take place in the solid state. Moreover, the thermal analysis demonstrated that the CUR and QU are stable in the nanoemulsion production temperature range. The proposed analytical method for the simultaneous quantification of the two actives was selective and linear for both compounds in the range of 0.5–12.5 µg/mL (R^2^ > 0.9997), precise (RSD below 3%), robust and accurate (recovery 100 ± 5 %). The method was validated in accordance with ICH Q2 R1 “Validation of Analytical Procedures” and CDER-FDA “Validation of chromatographic methods” guideline. Furthermore, the low limit of detection (LOD 0.005 µg/mL for CUR and 0.14 µg/mL for QU) and the low limit of quantification (LOQ 0.017 µg/mL for CUR and 0.48 µg/mL for QU) of the method were suitable for the application to drug release and permeation studies planned for the development of the nanoemulsions. The method was then applied for the determination of nanoemulsions CUR and QU encapsulation efficiencies (> 99%), as well as for the stability studies of the two compounds in simulated biological fluids over time. The proposed method represents, to our knowledge, the only method for the simultaneous quantification of CUR and QU in nanoemulsions.

## 1. Introduction

Quercetin (QU) and curcumin (CUR) are natural compounds that present interesting properties for the therapy of several diseases. QU is a common flavanol found in many vegetables, berries, fruits, beverages, and nuts [1]. QU has been found to decrease the inflammatory state induced by cholesterol oxidation products, a risk factor in neurodegenerative diseases, to lower the expression of pro-inflammatory cytokines, such as interleukin-6, tumor necrosis factor-α, interleukin-1b, and to inhibit the expression/activity of the cyclooxygenase-2 (COX-2) by suppressing the COX-2 mRNA expression [2]. As a consequence, QU is considered able to prevent neural damage [3,4]. However, despite the absorption by passive diffusion of QU across the intestine, the overall bioavailability of this compound is low and significantly variable among individuals [5].

CUR is a hydrophobic polyphenol derived from the rhizome of *Curcuma longa* that presents several medicinal properties similar to QU, such as anti-inflammatory, anti-oxidant, and anti-neurodegenerative effects [6,7]. Motaghinejad and collaborators demonstrated that CUR is able to slow down the progression of neuronal loss in adult male rats [8]. However, the most challenging drawbacks for its therapeutic use are the low water solubility, low bioavailability, and low blood concentrations after oral administration [9]. Taken together, the poor absorption from the gastrointestinal tract of CUR, its rapid metabolism in the liver and in the intestinal wall, and its limited blood brain barrier permeability are considered the main limitations to the therapeutic use of this compound in neurodegenerative disorders [10].

In fact, the role of oxidative stress and of the associated inflammation is becoming increasingly evident in the pathogenesis of neurodegenerative diseases [11,12,13]. The therapeutic potential of both CUR and QU is, however, further restricted by their poor stability in the physiological environment. The use of innovative nanotechnology-based formulations has been proposed in many studies with the aim of improving the bioavailability of natural compounds similar to CUR and QU [14,15,16]. Indeed, various nanocarrier systems have been demonstrated to provide interesting therapeutic benefits according to the nanocarrier properties, and the loaded pharmacologically active compound [17,18,19].

**Figure 1:**
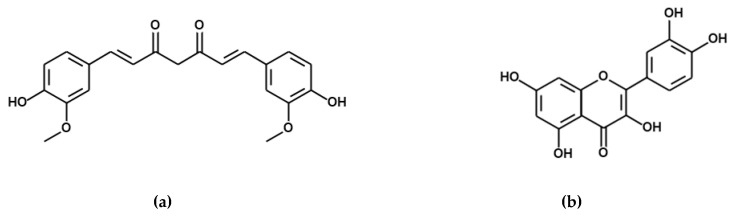
Chemical structures of curcumin (**a**) and quercetin (**b**).

In this sense, the development of nanocarriers loaded with CUR and QU in combination (Figure 1) and able to protect and direct these substances to the brain could represent an interesting approach for the treatment of neurodegenerative diseases. The association of these compounds can be highly advantageous providing an alternative to current pharmacological treatments since the two natural substances could act synergistically, and the use of these natural compounds could be economically advantageous in comparison to the development of new pharmacologically active chemical entities. However, despite the potential of these compounds in the treatment of neurodegenerative diseases, their association has been initially studied mainly to demonstrate their chemopreventive [20] and anticancer activity after oral administration [21]. A number of nanoformulations have been proposed in order to efficiently co-deliver curcumin and quercetin. Hyaluronan-containing niosomes were demonstrated to efficiently encapsulate both CUR and QU, to provide a controlled release of the two compounds and to provide a higher anti-inflammatory activity compared to a simple suspension after oral administration in the carrageenan-induced rat paw edema test [22]. In another study, CUR, QU and aspirin were co-encapsulated in chitosan nanoparticles obtained by ionotropic gelation with sodium hexametaphosphate obtaining relatively low encapsulation efficiencies (14–55%) but prolonged releases of the three drugs. The combination of the nanoencapsulated drugs was demonstrated to induce apoptosis and to stop the growth of human colon cancer cell line HCT116 more efficiently than the free chemopreventive drugs or two-drugs-loaded-nanoparticles [23]. Recently, the co-delivery of CUR and QU in Apoferritin nanoparticles of just 17 nm induced higher apoptosis in MCF-7 breast cancer cell line compared to the free combination of the drugs as a consequence of an improved cellular uptake of the drugs [24].

Therefore, the use of nanoemulsions containing both CUR and QU for the treatment of neurodegenerative diseases via the intranasal route makes the proposal of this work distinctive. In particular, for this approach polymer-based nanoparticles providing prolonged release don’t appear to be ideal due to the limited retention time of the formulation in the nasal cavity.

On the contrary, several studies indicate that nanoemulsions are a promising drug delivery system for nose-to-brain delivery. These nanocarriers are kinetically stable and not significantly affected by creaming, coalescence, flocculation or sedimentation during storage time [25]. The formulations are generally non-toxic and non-irritant being manufactured using low concentrations of surfactants safe for human consumption (generally recognized as safe, GRAS) [26]. In particular, nanoemulsion appears optimal nanocarriers for drugs with poor water solubility and/or presenting stability issues (hydrolysis, oxidation, and enzymatic degradation in physiologic conditions) and their large surface area enhances the permeation through biological barriers [27]. Therefore, considering the lipophilicity of CUR and QU, nanoemulsions appear particularly advantageous for the formulation and delivery into the brain of these natural compounds.

Nanoemulsion-based formulations provide several significant and unique advantages over conventional formulations, being favorable for drug delivery via several administration routes [28]. For example, Prasad and collaborators developed a lecithin-based curcumin-loaded nanoemulsion to enhance its anti-tumor efficacy through ultrasound-induced sonoporation of the cell membrane in the presence or absence of microbubbles (MB). The developed CUR nanoemulsions showed a hydrodynamic diameter of 60–120 nm and zeta potential values from −20 to −45 mV. The cytotoxicity of CUR nanoemulsion against MDA-MB-231 breast cancer cells and B16F10 melanoma cancer cells in the presence of ultrasound and MB was increased 100 and 64-fold, respectively, as a consequence of sonoporation-induced higher cell internalization. The in vivo pharmacokinetic study in rats evidenced also that the CUR nanoemulsion enhanced greatly the oral bioavailability of CUR in comparison to free curcumin, with an absolute bioavailability value of 78.61%. Furthermore, an in vivo study in C57BL/6 mice bearing B16F10 melanoma subcutaneous tumor on both flanks was carried out to evaluate the in vivo anti-tumor efficacy of this approach. The animals were treated orally with free CUR or CUR-loaded nanoemulsion (40 mg/kg), and after the injection of sulfur hexafluoride (SF6)-containing-lipid-shelled-microbubbles (MB) via the tail vein, the ultrasound treatment was applied only to the tumors developed on the right flank. The application of ultrasounds actually reduced the tumor volume and relative growth rate of the tumor. This study showed that the nanoemulsion was able to protect the CUR and that the association of the CUR nanoemulsion with ultrasound has the potential to target and increase the drug’s accumulation in the tumor and enable effective antitumor treatment [29].

Another study aiming to improve the topical delivery of imiquimod (IMQ) and CUR using a nanoemulsion-based delivery system was performed by Algahtani and collaborators. IMQ is a topical immunomodulator used in the treatment of superficial basal cell carcinoma [30]. A low-energy emulsification method was used to prepare nanoemulsions loaded with IMQ and CUR. It was shown that fatty acids, such as oleic acid, instead of fatty acid esters should be used as the oil phase for the design of IMQ containing nanoemulsion as the acid improve IMQ solubility. The mean droplet size of the optimized nanoemulsion was 76.93 nm (PDI 0.121) and zeta potential −20.5 mV. A nanoemulgel delivery system was prepared by dispersing the CUR and IMQ-loaded nanoemulsion uniformly in a Carbopol 934 hydrogel. The nanoemulgel exhibited a significant improvement of skin permeability and deposition profile after topical application. The in vivo effectiveness of the combination of these two active compounds in a nanoemulgel system was compared to the IMQ nanoemulgel and IMQ gel formulation through topical application for 10 days in BALB/c mice. The combination of CUR with IMQ in the nanoemulgel system prevented the appearance of psoriasis-like symptoms, a reaction often associated with the topical use of imiquimod, compared to the other tested formulations [31].

A study focused on the development of a nanoemulsion containing QU was performed by Kaur and collaborators. The sonication method was used to prepare a QU loaded o/w nanoemulsion using saponin and Tween 80 as surfactants and a mixture of lemon oil and corn oil as the oil phase. The obtained formulation of QU using only saponin as a surfactant had an average particle size of 52 ± 10 nm and zeta potential of −41 ± 8 mV. The developed QU-loaded nanoemulsion was demonstrated to be able to protect QU from hydrolysis, UV light degradation and the free radical scavenging activity showed that the QU nanoemulsion presents the highest antioxidant activity compared to nanoemulsions loaded with curcumin or ascorbic acid [32].

Based on the data presented in the literature, nanoemulsions could help protect CUR and QU from degradation and contribute to the control of the release of these compounds. Hence, this paper reports the pre-formulation studies and analytical method validation for the development of a CUR/QU nanoemulsion, as the first step towards a new treatment of neurodegenerative disorders with the combination of these two compounds. The nanoemulsion was developed using castor oil and purified fish oil (DHA/EPA) as the oil phase, PEG 660-stearate as surfactant, and egg lecithin as co-surfactant. The castor oil is a long-chain triglyceride oil used in pharmaceutical formulations as solubilizing excipient [33], and purified fish oil was demonstrated to provide enhanced neuroprotection in association with QU in rats [34].

In this sense, the development and characterization of this new pharmaceutical formulation required the evaluation of parameters such as drug content and stability of the nanoencapsulated compounds in comparison to free CUR and QU. The prepared nanoemulsions were characterized in terms of size, zeta potential, and PDI over time (up to 30 days) at different temperatures (4, 25, and 40 °C). Thus, in this work, a simple, sensitive, and specific high-pressure liquid chromatography (HPLC) method was developed and validated for the separation and simultaneous quantification of CUR and QU in a nanoemulsion. To evaluate the stability of CUR and QU, the validated analytical method was applied to monitor the content of the active ingredients during a stability study comparing the compound stability in a free and nanoencapsulated form.

## 2. Materials and Methods

### 2.1. Materials for Analytical Method Development

Acetonitrile and methanol of HPLC grade used in the analysis were purchased from Panreac^®^ (Barcelona, Spain). Ultrapure water was obtained from a Milli-Q water system (Millipore^®^, Burlington, MA, USA). Phosphoric acid (Reagen^®^, Rio de Janeiro, Brazil) used in the study was of analytical reagent grade.

### 2.2. Materials for Nanoemulsion Production and Characterization

CUR, QU, PEG 660-stearate, and castor oil were purchased from Sigma-Aldrich (St. Louis, MO, USA). Egg lecithin (Lipoid E80^®^) and Purified Fish Oil (DHA/EPA) were purchased from Lipoid (Steinhausen, Switzerland). Polyethylene glycol 400 (PEG400) was purchased from Synth (São Paulo, Brazil). Ultrafree-MC^®^ centrifugal filtration devices (10,000 Da MWCO) were purchased from Millipore^®^. Uranyl acetate was purchased from Electron Microscopy Sciences (Hatfield, PA, USA) All other reagents, where not specified otherwise, were of analytical grade.

### 2.3. Compatibility Study of Curcumin and Quercetin

#### 2.3.1. Preparation of Curcumin/Quercetin Binary Mixtures

The binary mixtures were prepared and mechanically homogenized with a mortar and pestle by taking CUR and QU in a 1:1 proportion by weight. These mixtures were further used for X-ray powder diffraction and thermal analyses.

#### 2.3.2. Thermal Analyses

Thermogravimetric analysis (TGA) was performed using a TGA-50 instrument (Shimadzu, Kyoto, Japan) under a nitrogen atmosphere with a flow rate of 50 mL/min at a heating rate of 10 °C/min over the range from 0 to 900 °C and using approximately 3 mg of sample in a platinum cell.

DSC data were collected on a DSC-60 instrument (Shimadzu). Approximately 1 mg samples were placed in aluminum pans, and the temperature ramp was set to increase from 50 to 225 °C with a heating rate of 10 °C/min under nitrogen flow (50 mL/min).

#### 2.3.3. X-ray Powder Diffraction (PXRD)

PXRD patterns were collected on a D8 Advance instrument (Bruker, Billerica, MA, USA) operating at 1.5418 Å, 40 kV voltage, and a current of 40 mA using a Cu K-α radiation source. The samples were contained in a sample holder and the data acquisition was done in a 2 θ range from 2 to 70 degrees at 0.05 degrees every 2 s step size over a total period of 50 min.

#### 2.3.4. Equipment and Chromatographic Conditions

Chromatographic analyses were performed on a Flexar HPLC system (Perkin Elmer Inc., Waltham, MA, USA) equipped with a quaternary pump, photodiode array detector, and automatic injection with a 15 µL sample loop. The chromatographic separations were performed using a 150 × 4.6 mm i.d., 5 µm particle size, C18 column (Zorbax ODS, Agilent Technologies, Wilmington, DE, USA) protected by a C18 guard column (150 µm, 140 Å, Phenomenex, Torrance, CA, USA) in gradient elution mode with a mobile phase obtained mixing aqueous phosphoric acid 1% *w*/*v* adjusted at pH 2.6 (Eluent A) and acetonitrile (Eluent B) at a flow rate of 1.0 mL/min (Table 1). The detection wavelength was set at 400 nm and the oven temperature was maintained at 40 ± 1 °C during the whole analysis time.

#### 2.3.5. Preparation of Stock and Working Solutions

Standard stock solutions of CUR and QU were freshly prepared by dissolving the compounds in methanol (1 mg/mL). Calibration curves were prepared using working solutions with concentration values 0.25, 0.5, 1.0, 2.5, 5.0, 7.5, 10.0 and 12.5 µg/mL by diluting the stock solution in a standard diluent obtained by the binary mixture (50:50, *v*/*v*) of methanol and 1% phosphoric acid pH 2.6. An aliquot (15 µL) of each working solution was then directly injected into the HPLC for further analysis.

#### 2.3.6. HPLC Method Validation

The proposed HPLC method was validated under the optimized conditions regarding its linearity range, selectivity and system suitability, sensitivity, precision, accuracy, robustness, and stability of the assay according to the analytical methods validation requirements. The method was validated in accordance with the ICH Q2 R1 (Validation of Analytical Procedures: Text and Methodology) [35] and CDER-FDA guidelines (Validation of chromatographic methods) [36].

##### Linearity Range

The linearity range was evaluated by measuring the chromatographic peak area responses of the compounds at seven concentration levels and in triplicate. Calibration curves were constructed by plotting the peak area against the concentration of CUR and QU, which then were interpolated by linear regression.

##### Selectivity and System Suitability

To ensure the selectivity of the proposed method, a drug-free nanoemulsion was prepared and analyzed in the described chromatographic conditions. Subsequently, the chromatographic separation of the two analytes was evaluated using the highest work solution (12.5 μg/mL CUR/QU) to determine the number of theoretical plates (*N*), analytes retention factor (*K’*), selectivity (α), symmetry (*T*), and to calculate peaks resolution (*Rs*) and area repeatability (calculated using the response relative standard deviation, RSD):

(1) $$N\;=\;5.55\;x\;{\left(\frac{t_{r}}{W_{h}}\right)}^{2}$$

(2) $$K\prime \;=\;\frac{t_{r}-t_{0}}{t_{0}}=\;\frac{t\prime_{r}}{t_{0}}$$

(3) $$\alpha \;=\;\frac{t\prime_{rB}}{t\prime_{rA}}=\;\frac{t_{rB}-t_{0}}{t_{rA}-t_{0}}=\;\frac{K\prime_{B}}{K\prime_{A}}$$

(4) $$T=\;\frac{W_{0.05}}{2f}$$

(5) $$Rs\;=\;\frac{2.(t_{rA}-t_{rB})}{\left(W_{b}^{A}+W_{b}^{B}\right)}$$

where *t_r_* = retention time, *t*_0_ = dead time, *W_h_* = width at average peak height, *W_b_* = width at the base of the peak, *W*_0.05_ = width at 5% of the peak height and f = width of the anterior portion of the peak at 5% of the height

##### Sensitivity

The sensitivity was determined by means of the limit of detection (*LOD*) and limit of quantification (*LOQ*) [35]. One of the ways to calculate the LOD (Equation (5)) and LOQ (Equation (6)) is based on the standard deviation (σ) of the y-intercepts and slope (s) obtained from the equation obtained by linear regression of the calibration standards:

(6) $$LOD\;=\;3.3\cdot \frac{\sigma }{s}$$

(7) $$LOQ\;=\;10\cdot \frac{\sigma }{s}$$

##### Precision and Accuracy

The accuracy and precision of the method were estimated by sextuplicate quality control (QC) samples prepared using the standard diluent mixture (methanol:1% phosphoric acid pH 2.6) at the following concentrations 0.5 µg/mL (low QC), 2.5 µg/mL (medium QC), and 12.5 µg/mL (high QC) for both CUR and QU. Accuracy was established through back-calculation and expressed as the percent difference between the found and the nominal concentration for each compound, and the precision was calculated as the coefficient of variation (CV) of the replicate measurements. Calibration standards and QC samples were analyzed in three different batches in order to determine the intra and inter-batch variability. The intra-day precision (repeatability) was carried out by performing six consecutive analyses of standard solution at three different concentrations for each drug on the same day. The samples were also analyzed on different days to evaluate the inter-day precision (repeatability). The obtained values were evaluated through the dispersion of the results by calculating the standard deviation of the measurement series.

##### Robustness

The robustness of an analytical method is a measure of its capacity to resist changes due to small variations in parameter conditions. In this way, the method robustness was assessed as a function of changing the column temperature, mobile phase composition, and pH (Table 2). The time in which the variations of the mobile phase occurred were in accordance with the data in Table 1.

##### Stability

Two different evaluations were made to determinate the stability of the compounds. Firstly the stability of the CUR and QU in solution (50:50, methanol:1% phosphoric acid, *v*/*v*; pH 2.6), was investigated after storage for 7, 15, and 30 days under refrigeration (4 °C), and at room temperature (25 °C) using a methanolic solution of 2.5 µg/mL of each compound as a control. In a second step, the comparison between the stability of the free CUR and QU and the nanoencapsulated compounds were evaluated during 4 h in phosphate buffer (phosphate buffered saline, PBS pH 7.4:PEG 400 90:10 *v*/*v*) at 25 °C and 37 °C.

#### 2.3.7. Application of the Method

##### Nanoemulsion Preparation

After the development of a simple, accurate and precise method, the nanoemulsion containing CUR and QU (CQ NE) was produced. The nanoemulsion was composed of egg lecithin, castor oil and purified fish oil (DHA/EPA), PEG 660-stearate and water. The nanoemulsion was formed via high-energy emulsification followed by high-pressure homogenization of a mixture of a water phase and an oil phase. To prepare the nanoemulsion loaded with CUR and QU, the oil and aqueous phases of the emulsion were firstly prepared separately. In order to prepare the water phase, the surfactant PEG 660-stearate was dissolved in ultrapure water (1.5% *w*/*v*). The oil phase containing castor oil, Lipoid^®^ Purified Fish Oil (DHA/EPA) and egg lecithin (Lipoid E80^®^) was maintained for 30 min at 68 °C under magnetic stirring at 1500 rpm. In the oil phase, the weight ratio between castor oil, and purified fish oil was 1:1, and between the surfactant egg lecithin and both oils 1:4. The aqueous phase (60 mL) heated to 80 °C under magnetic stirring at 1500 rpm for 2 min was then added to the oil phase. The oil phase represented 9% of the final weight of the formulation, being the weight ratio between the aqueous phase and the oil phase 10:1. After adding the aqueous phase to the oil phase, the dispersion was homogenized for 2 min using a mechanic high performance dispersing device (Ultraturrax TP 18/10 – 10N; IKA-Werke GmbH, Staufen, Germany) at 14,500 rpm for 2 min to form the pre-emulsion. Finally, the pre-emulsion was processed with a high-pressure homogenizer (PandaPLUS 2000 Laboratory Homogenizer, GEA Niro Soavi, Parma, Italy), 13 cycles of 20 s each at 1000 bar, totaling 4 min and 20 sec. For the preparation of CQ NE, the CUR and QU compounds (45 mg each) were added to the organic phase of the formulation and maintained under heating (68 °C) and stirring (1500 rpm) for 30 min (Table 3).

##### Size and Zeta Potential Measurements

The average hydrodynamic diameter, particle size distribution (as polydispersity index) and zeta potential of the NEs were determined by dynamic light scattering and laser doppler anemometry, respectively, using a Zetasizer Nano Series (Malvern Pananalytical, Malvern, UK). The particle size measurements were performed at 25 °C after appropriate dilution of the samples in distilled water (1:100, approximately 10 µL of nanoemulsion per mL). Each size analysis lasted 300 s and was performed with a detection angle of 90°. The hydrodynamic radius was determined according to Stokes- Einstein’s equation (Equation (7)):

(8) $$R=\frac{ĸBT}{6\pi \eta D}$$

where ĸ*B* = Boltzmann’s constant (J/K), *T* = temperature (in K), *D* = diffusion coefficient and *η* = viscosity of the medium, water in this case (0.89 cP at 25 °C)

For measurements of zeta potential, the samples were placed in the electrophoretic cell, where an alternating voltage of ±150 mV was applied. The zeta potential values were calculated as mean electrophoretic mobility values using Smoluchowski’s equation.

##### Determination of Curcumin and Quercetin Concentrations in the Nanoemulsion

The CUR and QU content (total concentration) in the nanocarrier suspension was calculated after determining the drug concentration compared to the methanolic standard solution (2.5 µg/mL) and was expressed in mg/mL of CUR and QU. To perform the determination of the drug content the nanoemulsion was appropriately diluted (200 times) with methanol and phosphoric acid 1% (50:50, *v*/*v*; pH 2.6) for the extraction of the drugs from the formulation matrix. The CUR and QU recovery was calculated as the percentage of the total drug concentration found in the nanocarrier suspension in relation to the initially added amount. The entrapment efficiency (%) was estimated indirectly as the difference between the total recovered amount of CUR and QU of the nanocarrier and that found in solution after eliminating the inner phase nanodroplets by ultrafiltration. The ultrafiltrate was obtained by an ultrafiltration/centrifugation method of an aliquot (500 µL) of the nanoemulsion using an Ultrafree-MC^®^ (10,000 Da MWCO, Millipore^®^) centrifugated at 10,000× *g* for 30 min (Sigma 3K30, Osterode am Harz, Germany). All samples were analyzed in triplicate.

##### Morphologic Evaluation

The morphology of nanoemulsion was investigated using a Transmission Electron Microscope (TEM) (JEOL 1400, Indianapolis, IN, USA). A drop of the nanoemulsion was diluted suitably (1000×) with ultrapure water, deposited on a copper grid coated with carbon followed by the addition of a negative stain (20 µL of uranyl acetate 2% *w*/*v* solution). After 20 min incubation at room temperature, excess liquid was carefully drained with a piece of filter paper and the samples were put into a desiccator overnight to completely eliminate the solvent. Images were captured using the TEM operated at 80 kV and 30,000× magnification.

### 2.4. Data Presentation and Statistical Analysis

Each experiment was conducted in triplicate, and the data are represented as mean and standard deviation Statistical analysis was conducted using analysis of variance (ANOVA), followed by a *post-hoc* Bonferroni test; a *p*-value less than 0.05 (*p* < 0.05) was considered statistically significant. All statistical analyses were performed using Prism software (ver. 8.4.3, Graph-Pad Inc., San Diego, CA, USA).

## 3. Results

### 3.1. Pre-Formulation Studies with Curcumin and Quercetin

**Figure 2:**
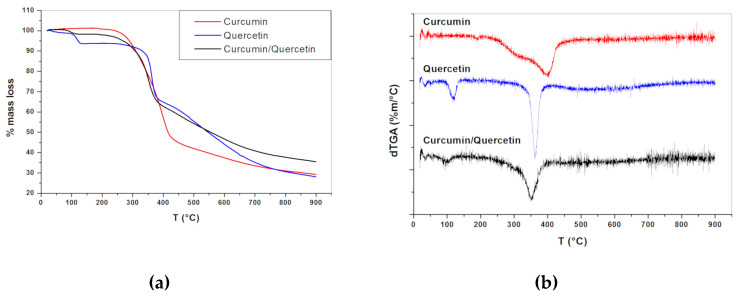
(**a**) Thermogravimetric analysis curves (TGA) and (**b**) first derivative of TGA curves (dTGA) obtained for curcumin, quercetin, and curcumin/quercetin binary mixture (1:1).

Thermogravimetric analyses of the two natural compounds and their mixture are presented in Figure 2. The TGA curves derivative (dTGA) (Figure 2b) shows that CUR was thermally stable up to 200 °C (T_onset_), when its thermal decomposition started. The decomposition ended at 427 °C. Concerning the mass loss, it was found that in this first step around 53% of the initial mass was lost. A second step of decomposition occurred in the range 428–900 °C, with a further mass loss of 17%. The residual mass at 900 °C was 30%.

In the case of QU, a first mass loss (around 6.5%) occurred between 94 and 137 °C (Figure 2a,b) and could be attributed to the loss of water of the sample. In fact, the percentage of water loss observed was very similar to the values reported in the literature [37,38,39]. This result highlights that QU in the solid-state shows a certain degree of hydration of their crystal lattice, despite its lipophilicity. According to Borghetti and co-workers, quercetin hydrates occur due to the intermolecular hydrogen bonds between hydration water molecules and hydroxyl groups of the flavonoid [38]. In this way, the presence of water molecules into the crystal lattice of the QU influences its molecular geometry and crystalline structure, leading to great changes in its thermal stability, solubility, and bioavailability. A second mass loss step started at 240 °C and ended at 385 °C, with a mass loss of 27.3%. A third mass loss occurred in the range 386–900 °C, with a mass loss of 38%. The residual mass at 900 °C was 28.2%. The TGA profile of the binary physical mixture of CUR and QU (1:1 ratio) (Figure 2a,b) showed three decomposition steps. The first mass loss corresponded to the release of water molecules due to the presence of QU hydrates in the mixture, the percentage mass loss was lower when compared to pure QU and occurred in the range of 72 to 127 °C (Figure 2a,b). The second and third mass losses correspond to the thermal decomposition of the compounds (CUR and QU) at the highest temperatures, as observed for pure compounds. Overall, the TGA profile of the binary mixture did not suggest significant interactions in the solid-state between the two compounds. Similar results were obtained by DSC analysis.

**Figure 3:**
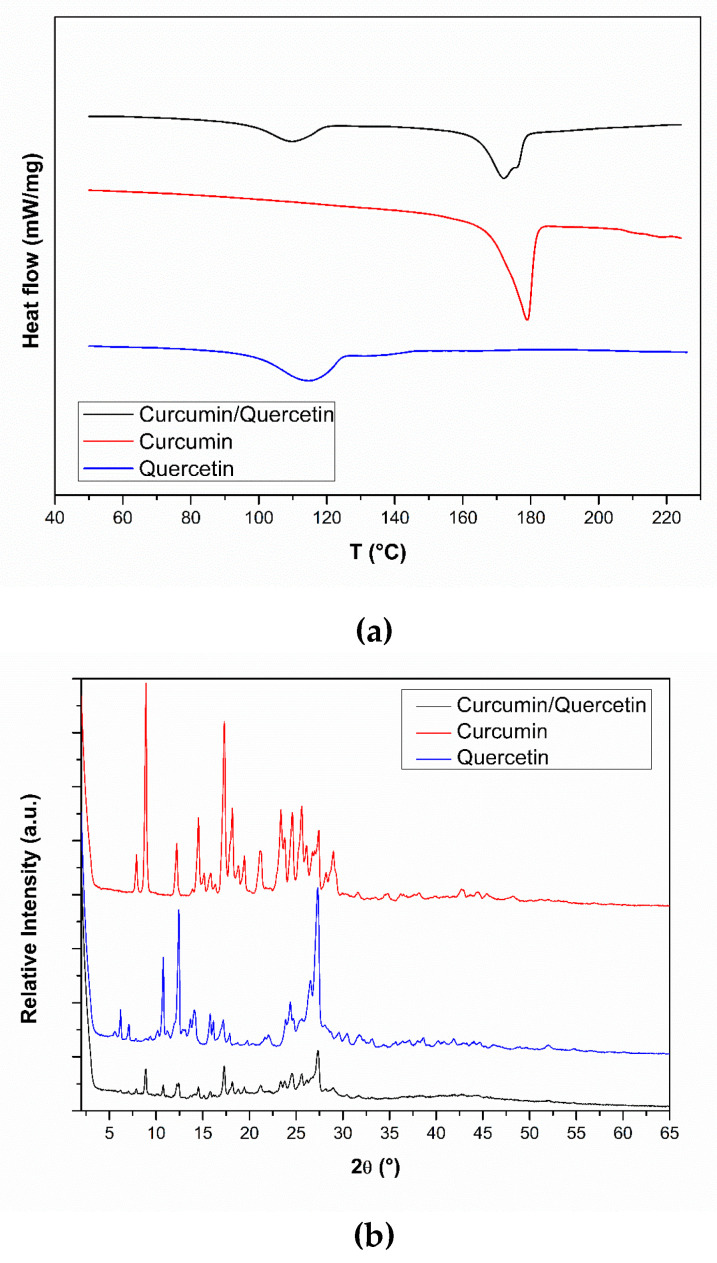
(**a**) Differential scanning calorimetry (DSC) profiles of curcumin, quercetin and the curcumin/quercetin binary mixture (1:1); (**b**) X-ray diffractograms of curcumin, quercetin and the curcumin/quercetin binary mixture (1:1).

The DSC curve for QU showed only one endothermic peak located in the range of 99 and 125 °C (maximum of the peak at 115 °C) correspondent to the release of water from the crystal lattice. The temperature of the maximum of the peak was much higher than the boiling point of water which means that molecules of water are strongly held by the QU crystals through hydrogen bonding [36]. The melting point of QU was not observed (316 °C) [40]. The CUR analysis showed only one endothermic peak in the range 172–183 °C (maximum of the peak at 176 °C), corresponding to the melting point of CUR reported in literature (175.1 °C) [41]. Furthermore, a shoulder was observed in the DSC curve from 200 °C. Similar thermal events observed to pure compounds were detected in the DSC curve for binary mixture, meaning that non-significant interactions occur between CUR and QU in the solid-state (Figure 3a). Interestingly, powder X-ray diffraction (PXRD) analysis conducted on the same materials showed that the diffraction of the binary mixture (Figure 3b) contained all the peaks of CUR and QU, with no marked displacement of the peaks being observed, indicating a lack of interaction in the physical mixture of the two compounds.

### 3.2. Method Development and Validation

**Figure 4:**
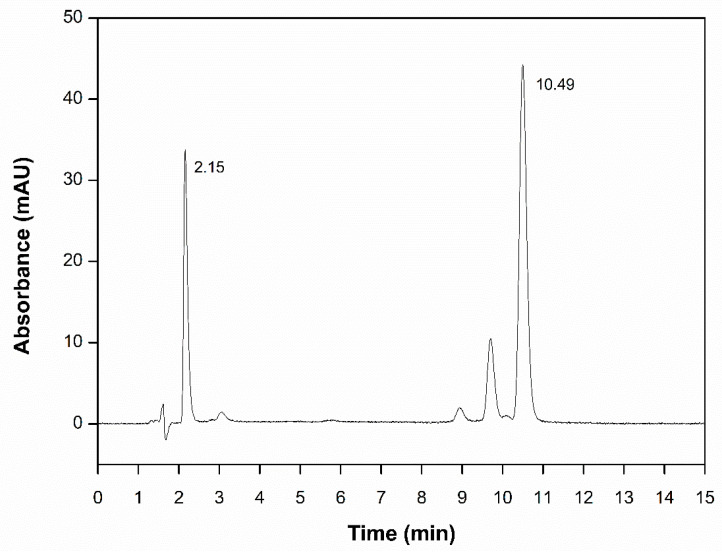
Chromatograms of the standard solution containing a binary mixture of curcumin and quercetin (12.5 µg/mL).

The best separation conditions for CUR and QU were achieved using a C_18_ analytical column in a gradient elution mode with a mobile phase composed of acetonitrile and a 1% phosphoric acid solution in water (Table 1), at a flow rate of 1.0 mL/min. The detection wavelength was set at 400 nm, and 40 ± 1 °C. A typical chromatogram is shown in Figure 4, with a retention time of 2.15 min being observed for QU, and 10.49 min for CUR. It was possible to observe three other peaks of non-interest in the chromatogram: an unidentified impurity (3.06 min) and the curcuminoids bisdemethoxycurcumin (8.94 min) and demethoxycurcumin (9.71 min) [42]. CUR being a natural product presents a number of impurities since the extract from the turmeric rhizome is rich in many curcuminoids, i.e., a mixture of CUR, bisdemethoxycurcumin, and demethoxycurcumin [43]. However, none of them appear to interfere with the elution and separation of the investigated analytes.

To evaluate the linearity of the method, calibration standards of CUR (0.25–12.5 µg/mL) and QU (0.25–12.5 µg/mL) were analyzed. A linear relationship was established for the injected concentration ranges versus the peak area for both analytes, with determination coefficients greater than 0.9997. Some validation parameters obtained with the calibration standards are reported in Table 4, while the linearity parameters of the method shown in Table 5.

The method’s selectivity was confirmed by the absence of interferences at the retention times of CUR and QU in the chromatograms obtained when the nanoemulsion prepared without the drugs (data not shown).

The analytical method was suitable for the separation, detection and quantification of analytes, as shown on Table 6, with peaks presenting good shape (QU - K’ 0.47; CUR - K’ 6.18), symmetry (QU - T 0.968; CUR - T 0.909), α 13.14 and resolution Rs 10.83. The system suitability of the developed method for QU and CUR showed a high value of resolution (Rs > 2, CDER-FDA acceptance criteria) [36], and the repeatability of peak area (RSD ≤ 1%), as reported on Table 6. The intra- and inter-day precision relative standard deviation (RSD%) was between 0.8 and 5.9 for CUR and 0.4 and 7.6 for QU. The recovery of the drugs was in the range of 99.46–101.63% with RSDs below 4.2% for CUR and in the range of 93.60–103.73% with RSDs below 4.64% for QU. The results are presented in Table 7.

In order to evaluate the robustness of the chromatographic method, assays were carried out by analyzing standard solutions (2.5 µg/mL) under slight variations of the method conditions, including column temperature, mobile phase composition and pH. The results from the robustness testing are displayed in Table 8.

**Figure 5:**
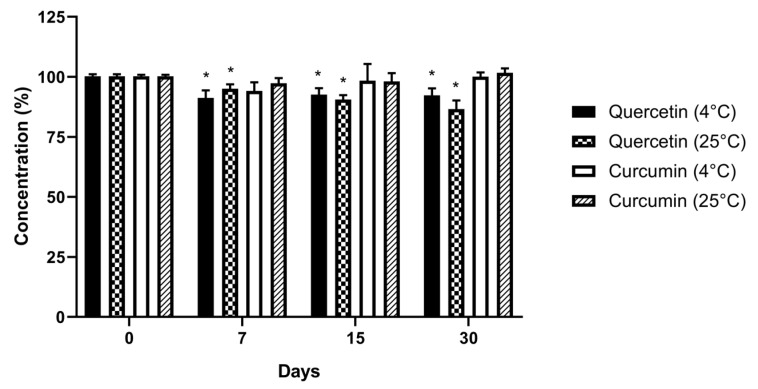
Data related to the stability of the standard methanolic solutions of CUR and QU (2.5 µg/mL) at 4 °C and 25 °C. N = 3. * *p* < 0.05.

Analyses in the concentration of CUR and QU in the stability test in the standard methanolic solution over time showed that the percent recovery of CUR and QU were, respectively, 94.1 ± 3.6% and 91.2 ± 3.1% under refrigeration (4 °C), and 98.1 ± 3.4% and 86.5 ± 3.6% at room temperature (25 °C) (Figure 5).

**Figure 6:**
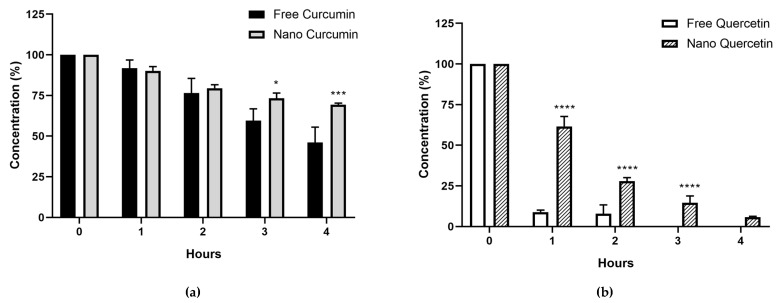
Data related to the stability of the free and nanoencapsulated CUR (**a**) and QU (**b**) in PBS:PEG400 (90:10) pH 7.4. N = 3 at temperature (37 °C). * *p* < 0.05, *** *p* < 0.001, **** *p* < 0.0001.

The evaluation of the stability of the free CUR and QU and the nanoencapsulated compounds up to 4 h in 90:10 PBS:PEG 400, *v*/*v*; pH 7.4, at 37 °C the percentage of the free form of CUR was 46.10 ± 9.4% within 4 h of evaluation and no QU was detected after 3 h of experiment. Conversely, the results of the nanoencapsulated compounds showed that after 4 h of experiment at 37 °C the percentage of the CUR and QU were found to be higher, respectively 69.33 ± 1.0% and 5.80 ± 0.5% (Figure 6).

### 3.3. Size, Polydispersity Index, and Zeta Potential

**Figure 7:**
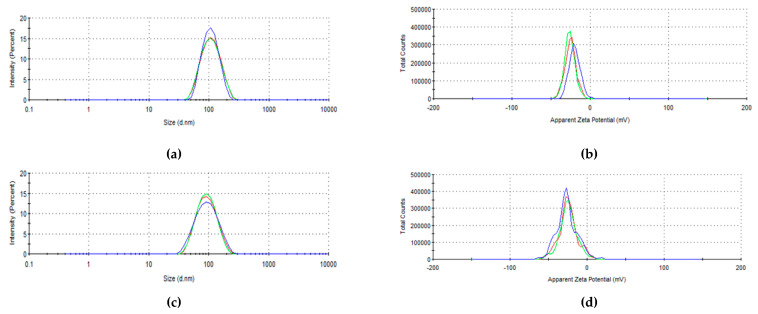
Size and surface charge distribution of CUR and QU-loaded nanoemulsions (panel (**a**) and (**b**)) and of control nanoemulsion prepared without the active compounds (panel (**c**) and (**d**)) (measurements performed immediately after production, day 0, at 25 °C).

The formulations containing CUR and QU showed an average diameter of 112.33 ± 1.51 nm, PDI of 0.101 ± 0.02 nm and zeta potential of −25.37 ± 0.51 mV, and the control nanoemulsion prepared without the active compounds showed a size of 98.11 ± 1.60 nm, PDI of 0.127 ± 0.01 nm and zeta potential of -25.10 ± 0.80 mV (see also Figure 7).

The evaluation of the size, PDI, and zeta potential during 30 days of storage at different temperatures (4, 25, and 40 °C) demonstrated that the formulation was stable in size and PDI (see Supplementary Material Figure S1) with values around 115 nm and PDI values consistently below 0.2, while showing a decrease in the zeta potential value from –25 to –40 mV, possibly attributable to a slight hydrolysis of phospholipids over time leading to a higher negative surface charge (Figure S2).

### 3.4. Determination of the Concentration of Curcumin and Quercetin in Nanoemulsion Using HPLC Analyses

The formulations containing CUR and QU showed an amount of 0.71 ± 0.08 mg/mL of QU, and 0.62 ± 0.08 mg/mL of CUR, and demonstrated that it was possible to encapsulate 94.66 ± 10.6% of QU in the developed nanocarriers and 82.66 ± 10.6% of CUR with an entrapment efficiency of > 99% for both compounds.

### 3.5. Transmission Electron Microscopy

**Figure 8:**
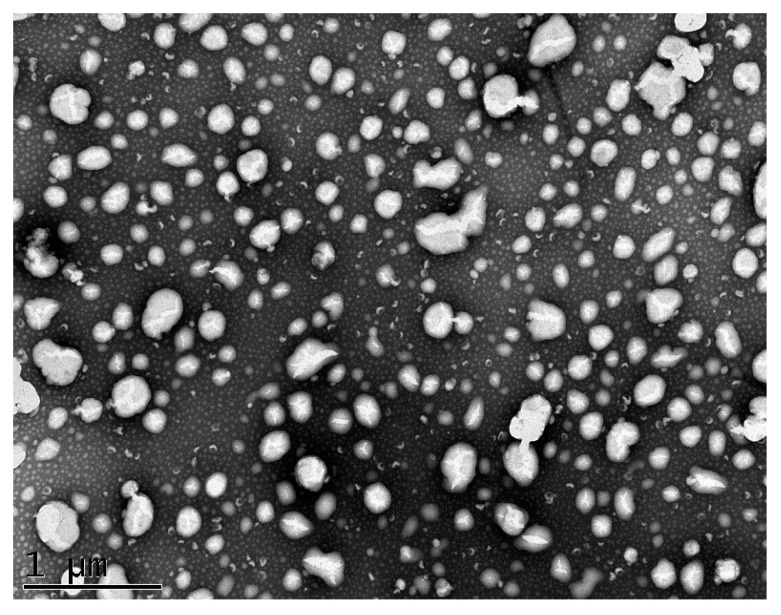
Transmission electron micrographs of nanoemulsion with curcumin and quercetin (CQ NE).

The TEM micrographs recorded using negative staining illustrated the morphology and size of the nanoemulsion produced (Figure 8). The nanocarriers appeared spherical in shape with size and particle size distribution in good accordance with the results of the analyses performed by dynamic light scattering. Moreover, the nanocarriers were well dispersed without significant agglomeration or morphological variations.

## 4. Discussion

Although advances in nanometric-sized formulations have opened the way to a new class of diagnostic and therapeutic nanomedicines for many diseases, their transfer from the benchtop research to currently marketed products are still limited. This lack of conversion is mostly attributed to problems with nanomedicines characterization, including physicochemical analyses, evaluation of their interaction with the biological environment and, often, the development of suitable analytical methods for encapsulated drug separation and determination [44].

In this study, the physicochemical properties of both active ingredients as well of their association were assessed using different techniques such as thermal analyses (TGA and DSC) and powder X-ray diffraction (PXRD).

Initially, the active ingredients and their combination were characterized using thermal analyses, which offer the ability to quickly screen for potential drug–drug incompatibilities. Such interactions can be of physical or chemical nature and may affect the stability and bioavailability of the final product, compromising the therapeutic efficacy and safety [45].

The TGA curve of CUR indicated that the thermal decomposition occurred in two steps, while no water was present in the sample. However, the decomposition for QU was observed to happen in three steps and, contrarily of CUR, the QU presents water molecules in the crystal lattice, influencing its molecular geometry and crystalline structure and affecting its thermal stability, solubility, and bioavailability. The profile of the binary mixture decomposition was very similar to the pure compounds, meaning that the physical mixture of CUR and QU (1:1 ratio) does not lead to significant interactions in the solid state and the compounds undergo thermal degradation simultaneously and independently.

The DSC technique was employed to analyze the occurrence of physicochemical events related to the thermal behavior and possible interactions between the compounds [46]. It is noteworthy that although such analyses were conducted upon heating the sample to high temperatures, which is not consistent with the process of nanoemulsion production, neither with its administration to patients, they afford important information regarding the physical properties of the samples [45]. The endothermic peak observed in the QU DSC curve can be attributed to the moisture of this compound and is in agreement with the TGA result as soon as the melting point of QU occurs at 316 °C. However, the endothermic peak observed in the CUR DSC curve corresponds to the melting point of CUR, and the shoulder that is observed in the DSC curve from 200 °C in the CUR curve corresponds to the beginning of the decomposition of this compound, as seen in the TGA curve.

The PXRD analyses of the binary mixture contained virtually all the peaks of CUR and QU, with no marked displacement of the peaks, and no appearance of any new peaks was observed, which means that if exist some interaction between the compounds, this one is probably not strong enough to take place in the solid state.

Chromatographic method development and validation play an important role in the design, development and manufacture of nanosized pharmaceuticals due to their ability to separate and quantify several analytes of interest from the components of the nanocarriers. Indeed, the quantification of the pharmaceutically active compounds is required for the study of the nanosystems encapsulation efficiency, drug release kinetics, as well as stability and even for the investigation of their interaction with biological interfaces [47].

Considering the unique properties showed by nanoemulsion, in the present work, CUR and QU, castor oil and DHA, as well as egg lecithin composed the oil phase and Milli Q^®^ water and PEG stearate composed the aqueous phase. In this context, HPLC-UV was selected as an analytical tool for the simultaneous quantification of CUR and QU in the developed nanoemulsion through a rapid, simple, and gradient method. The method was validated in accordance with ICH Q2 R1 (Validation of Analytical Procedures: Text and Methodology) and CDER-FDA guideline (Validation of chromatographic methods). Analytes retention factor, asymmetry and number of theoretical plates (N > 2000) were investigated. All obtained values were in accordance with the required criteria, indicating the suitability of the analytical method. No other co-eluting peak was interfering with those of interest and the method was specific for the determination of CUR and QU. The alterations in the conditions of the chromatographic method to evaluate the robustness like, the temperature change, mobile phase composition and pH did not promote any significant variations in the retention time of CUR and QU peaks indicating that the method was robust. The developed and validated method demonstrated to efficiently determine CUR and QU loaded on nanoemulsion vectors. The extraction method of the drug proved to be efficient since the recovery range of the compounds from the nanoemulsion was between 99.46 and 101.63%.

The preparation method via high-energy emulsification followed by high-pressure homogenization was efficient to obtain the nanoemulsions. The zeta potential that is an important parameter to determine the stability of the formulations since values higher than |30| mV are considered ideal in terms of electrostatic stability. However, systems that have steric stabilizers such as PEG stearate do not follow this rule, being more stable with wider values of surface charge [48]. Zeta potential values lower than |30| mV, do not represent low stability for the system since a steric stability can be hypothesized [44]. The negative charge observed for the developed nanoemulsion (–25 mV) resulted from the presence of charged phospholipids such as phosphatidylserine, phosphatidylinositol, and phosphatidic acid in the lecithin phospholipids mixture [49]. The developed formulations showed an incorporation of high amounts of CUR (0.62 mg/mL) and QU (0.71 mg/mL) in the formulation. The encapsulation of the two natural compounds led to a slight increase in the average particle size of the nanoemulsion (98.11 vs. 112.33 nm) as often observed in the case of nanocarriers [50]. Generally, in the case of nanoemulsions this can be attributed to effects related to the dispersion of the inner phase in the bi-phasic system such as increase in the viscosity [51] or change at the droplet interface [52]. Considering the relatively low content of the two drugs, that is unlikely to have a relevant impact on the inner phase viscosity, an interference of the two natural compounds with the phospholipids layer stabilizing the nanodroplets is more likely to have occurred in reason of their polyphenolic and flavonolic structure.

The morphological analysis of the nanoemulsions carried out by TEM showed that the developed nanocarrier particles appeared spherical in shape with a size and polydispersity index in good accordance with the results of the analyses performed by dynamic light scattering.

The evaluation of the concentration of CUR and QU during 30 days showed no changes in the amount of the CUR in methanolic solution at 4 °C and 25 °C, however the One-way ANOVA detected statistically significant differences among the concentration of QU after 7 days at 4 °C and 25 °C compared to the initial amount of QU. The drugs in methanolic solution were stable for at least 15 days under storage conditions, with RSDs below 8%. The stability study of free CUR and QU at 25 °C in phosphate buffer (90:10, PBS:PEG 400, *v*/*v*; pH 7.4) showed a higher degradation of QU compared to CUR. However, the comparison between the free form of CUR and QU and the developed nanoemulsion loaded with the two natural compounds at 37 °C in phosphate buffer (90:10, PBS:PEG 400, *v*/*v*; pH 7.4) showed that the latter protected at least partially CUR and QU from the degradation.

## 5. Conclusions

The combination of CUR and QU in a pharmaceutical formulation is of great importance owing to the potential of generating a new option for the treatment of neurodegenerative diseases. In this sense, the study of formulation using different techniques such as TGA, DSC, and PXRD was essential to examine the existence of possible interactions between these two compounds (CUR and QU). Furthermore, an HPLC method was developed and validated according to standard guidelines, and it is the first reported method for the simultaneous determination of CUR and QU in a nanocarrier such as nanoemulsion. The possible interactions between CUR and QU demonstrated no strong enough interaction in the solid-state, and the thermal analysis demonstrated that CUR and QU are stable under the temperatures of the nanoemulsion production. The proposed method was selective and linear in the range of 0.5–12.5 µg/mL, precise, robust and accurate with no interfering peaks in the regions of interest. The low detection and quantification limits for CUR and QU obtained were suitable for the application of the method for the studies of drug entrapment efficiency and formulation stability with the developed nanoemulsion. Moreover, it was found that the CUR and QU were better stable into nanoemulsions in the simulated biological fluids, compared to the free material, which could favor a higher bioavailability and consequently drug effectiveness. The proposed o/w nanoemulsion appears to be a promising nanocarrier for the nose-to-brain delivery of the combination of CUR and QU. In order to develop this formulation for clinical use, several steps have to be taken such as: demonstration of pharmacological efficacy in relevant models of neurodegenerative diseases, selection of a suitable device for nasal administration and definition of the optimal conditions of storage for product stability.

## Figures and Tables

**Table 1:** Chromatographic conditions of the gradient HPLC analytical method.

Time (min)	Eluent A (%)	Eluent B (%)	Flow Rate (mL/min)
0.01	60	40	1.0
5.00	60	40	1.0
6.00	50	50	1.0
15.00	50	50	1.0

Eluent A: Phosphoric acid 1% *w*/*v* in water, pH 2.6; Eluent B: Acetonitrile

**Table 2:** Analytical parameters and their levels of changes used in the robustness test of the method for CUR and QU by HPLC.

Parameter	Condition
Column temperature	35 °C
	40 °C
	45 °C
Mobile phase composition (aqueous phosphoric acid 1%:acetonitrile)	35:65/45:55
	40:60/50:50
	45:55/55:45
pH	2.3
	2.6
	2.9

**Table 3:** Composition of the nanoemulsion as prepared through the high-pressure homogenization method.

Formulation	PEG 660-Stearate (%; *w*/*v*) *	Castor Oil (mg)	Purified Fish Oil (mg)	Egg Lecithin (mg)	CUR (mg/mL)	QU (mg/mL)
CQ NE	1.5	2400	2400	1200	0.75	0.75
NE	1.5	2400	2400	1200	-	-

* aqueous phase (60 mL)

**Table 4:** Data related to the LOD, LOQ, slope, and interception for curcumin and quercetin HPLC analysis method.

	Parameters	Validation Results
Curcumin	Linearity	Calibration range (µg/mL): 0.5–12.5
		y = 40,565x − 272.07 (R^2^ = 0.9998)
	LOD	0.005 µg/mL
	LOQ	0.017 µg/mL
	Slope	40,565 ± 201
	Intercept	−272.07 ± 1246.54
Quercetin	Linearity	Calibration range (µg/mL): 0.5–12.5
		y = 18,043x + 129.07 (R^2^ = 0.9997)
	LOD	0.14 µg/mL
	LOQ	0.48 µg/mL
	Slope	18,043 ± 118
	Intercept	129.07 ± 733.51

**Table 5:** Data related to the linearity of the developed HPLC method with its respective average, precision, and accuracy.

Concentration (µg/mL)	Curcumin			Quercetin		
	Concentration Found (µg/mL)	Accuracy (%)	Precision (%)	Concentration Found (µg/mL)	Accuracy (%)	Precision (%)
0.5	0.497 ± 0.005	99.40 ± 1.10	1.20	0.518 ± 0.012	103.60 ± 2.54	2.69
1.0	1.024 ± 0.008	102.40 ± 0.85	0.87	1.008 ± 0.012	100.80 ± 1.85	2.08
2.5	2.503 ± 0.014	100.12 ± 0.56	0.59	2.501 ± 0.020	100.04 ± 0.88	0.87
5.0	5.005 ± 0.049	100.10 ± 0.98	0.99	4.993 ± 0.020	99.86 ± 0.40	0.40
7.5	7.517 ± 0.023	100.22 ± 0.30	0.30	7.489 ± 0.074	99.85 ± 0.99	1.10
10.0	10.019 ± 0.019	100.19 ± 0.19	0.20	9.982 ± 0.030	99.82 ± 0.30	0.34
12.5	12.579 ± 0.090	100.63 ± 0.76	0.86	12.496 ± 0.062	99.96 ± 0.49	0.53

**Table 6:** System suitability chromatographic parameters.

Analyte	N	K´	T	α	Rs
QU	3506	0.47	0.968	-	-
CUR	20774	6.18	0.909	13.14_QU/CUR_	10.83_QU/CUR_

Abbreviations: N, number of theoretical plates; K’, retention factor; T, symmetry; α, selectivity; Rs, resolution; QU, quercetin; BDCUR, bisdemethoxycurcumin; DCUR; demethoxycurcumin; CUR, curcumin.

**Table 7:** Data related to the repeatability and intermediate precision of the developed HPLC method.

Samples	Intra-Day Precision (Repeatability)			Inter-Day Precision (Repeatability)		
**Curcumin** **(µg/mL)**	**Concentration Found (µg/mL)**	**Accuracy** **(%)**	**Precision** **(%)**	**Concentration Found (µg/mL)**	**Accuracy** **(%)**	**Precision** **(%)**
0.5	0.50	101.63 ± 4.2	5.9	0.49	99.46 ± 1.10	1.2
2.5	2.49	99.98 ± 1.0	1.5	2.50	100.12 ± 0.56	0.5
12.5	12.65	101.26 ± 1.0	1.4	12.57	100.63 ± 0.76	0.8
**Quercetin** **(µg/mL)**	**Concentration Found (µg/mL)**	**Accuracy** **(%)**	**Precision** **(%)**	**Concentration Found (µg/mL)**	**Accuracy** **(%)**	**Precision** **(%)**
0.5	0.46	93.60 ± 4.64	7.6	0.51	103.73 ± 2.54	2.6
2.5	2.46	98.68 ± 1.84	3.2	2.50	100.06 ± 0.88	0.8
12.5	12.43	99.44 ± 0.67	1.1	12.49	99.97 ± 0.49	0.4

**Table 8:** Results obtained from the study of robustness of the HPLC method.

Variable	Value	Curcumin Content ^a^ (μg/mL)	RSD ^a^ (%)	Quercetin Content ^a^ (μg/mL)	RSD ^a^ (%)
Column temperature (°C)	35	2.53 ± 0.04	1.67	2.54 ± 0.02	0.82
	40	2.50 ± 0.03	1.59	2.50 ± 0.03	1.87
	45	2.51 ± 0.03	1.41	2.49 ± 0.01	0.40
Mobile phase composition (1% phosphoric acid:acetonitrile, *v*/*v*; pH 2.6)	35:65/45:55	2.51 ± 0.06	2.39	2.51 ± 0.06	2.58
	40:60/50:50	2.50 ± 0.03	1.59	2.50 ± 0.03	1.87
	45:55/55:45	2.51 ± 0.03	1.31	2.54 ± 0.05	2.09
pH of the mobile phase	2.3	2.50 ± 0.02	0.89	2.49 ± 0.04	1.94
	2.6	2.50 ± 0.03	1.59	2.50 ± 0.03	1.87
	2.9	2.50 ± 0.03	1.25	2.49 ± 0.09	0.04

^a^ Mean of three replicates

## Supplementary Materials

The following are available online at https://www.mdpi.com/2079-4991/10/9/1650/s1, Figure S1: Evaluation of the size and PDI of CUR and QU-loaded nanoemulsion during 30 days of storage at different temperatures (4, 25, and 40 °C), Figure S2: Evaluation of the zeta potential of CUR and QU-loaded nanoemulsion during 30 days of storage at different temperatures (4, 25, and 40 °C).
