# Supplementary material for: Curcumin and Quercetin-Loaded Nanoemulsions: Physicochemical Compatibility Study and Validation of a Simultaneous Quantification Method
Source: Nanomaterials (Basel). 2020 Aug 22;10(9):1650. doi: 10.3390/nano10091650 (PMC7558409; doi:10.3390/nano10091650)
Supplement: Supplementary file 1 [file nanomaterials-10-01650-s001.pdf]

nanomaterials

Supplementary Material

Curcumin and Quercetin-Loaded Nanoemulsions: Physicochemical Compatibility Study and Validation of a Simultaneous Quantification Method

Gustavo Richter Vaz [1,2], Adryana Clementino [3], Juliana Bidone [4], Marcos Antonio Villetti [5], Mariana Falkembach [1], Matheus Batista [1], Paula Barros [1], Fabio Sonvico [3,*] and Cristiana Dora [1,*]

[1] Laboratório de Nanotecnologia Aplicada à Saúde, Programa de Pós-Graduação em Ciências da Saúde, Universidade Federal do Rio Grande, Rio Grande 96210-900 (RS) Brazil; richtervaz@gmail.com (G.R.V.) mari_falkembach@hotmail.com (M.F.); mbmatheus54@gmail.com (M.B.); alicebarros.pb@gmail.com (P.B.); cristianadora@gmail.com (C.D.)

[2] Coordenação de Aperfeiçoamento de Pessoal de Nível Superior (CAPES), Brasilia, 70040-020 (DF) Brazil

[3] Food and Drug Department, University of Parma, Parma 43124 (PR) Italy; adryanarc@gmail.com (A.C.); fabio.sonvico@unipr.it (F.S.)

[4] Centro de Ciências Químicas, Farmacêuticas e de Alimentos, Universidade Federal de Pelotas, Pelotas 96010-900 (RS) Brazil; julianabidone@gmail.com

[5] Laboratório de Espectroscopia e Polímeros, Departamento de Física, Universidade Federal de Santa Maria, Santa Maria 97105-900 (RS) Brasil; mvilletti@hotmail.com

* Correspondence: fabio.sonvico@unipr.it Tel.: +39 0521 906282 (F.S.); cristianadora@gmail.com (C.D.) Tel.: +55 53 32935313 (C.D.)

Received: 31 July 2020; Accepted: 21 August 2020; Published: date

Evaluation of the size, PDI, and zeta potential of CUR and QU-loaded nanoemulsion during 30 days of storage at different temperatures (4, 25, and 40 °C).

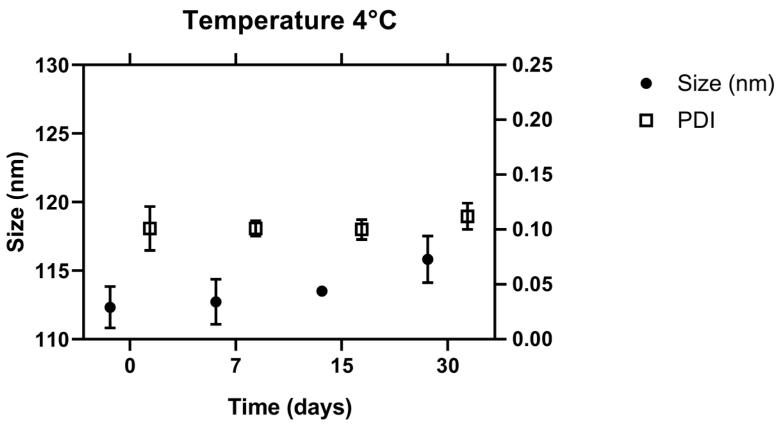

(a)

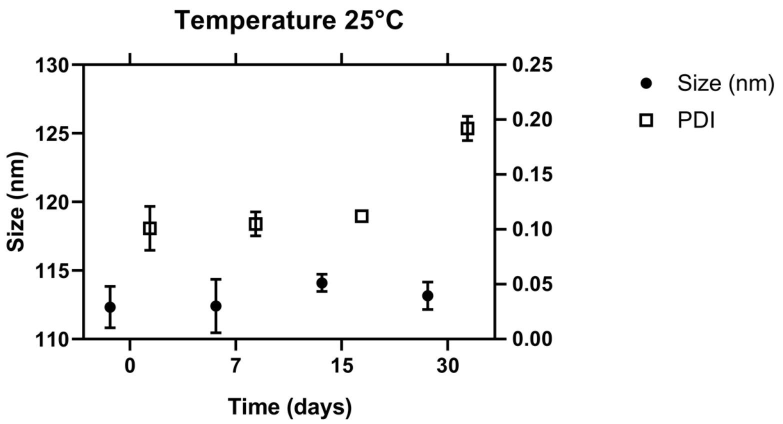

(b)

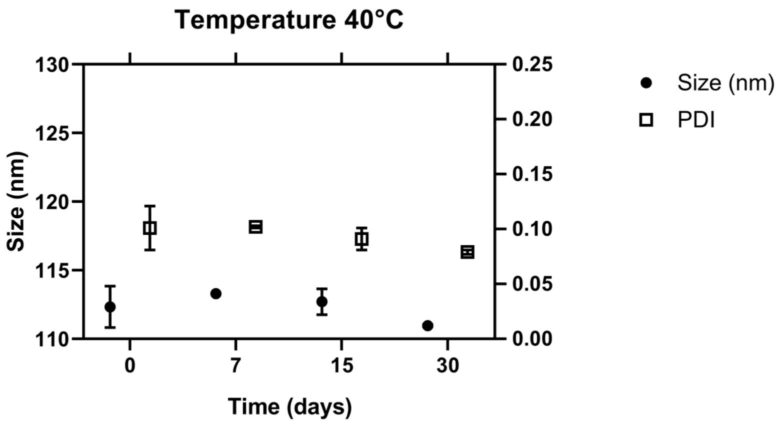

(c)

Figure S1. Data related to the evaluation of the size and PDI CUR and QU-loaded nanoemulsion during storage (30 days) at different temperatures 4°C **(a)**, 25 °C **(b)**, and 40 °C **(c)** (mean ± SD, n = 3).

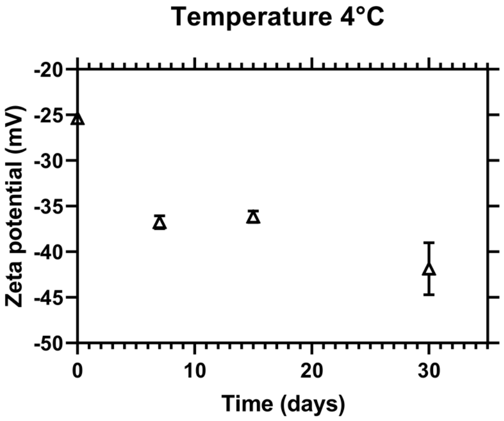

(a)

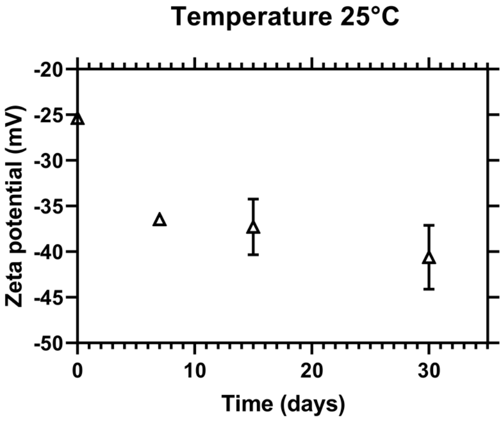

(b)

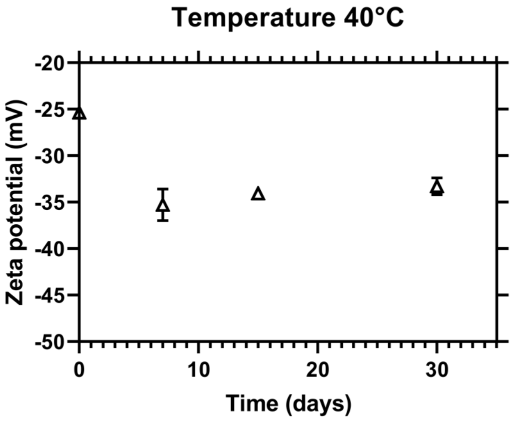

(c)

Figure S2. Zeta potential values of CUR and QU-loaded nanoemulsion during storage (30 days) at different temperatures 4 °C **(a)**, 25 °C **(b)**, and 40 °C **(c)** (mean ± SD, n = 3).

.
